# Infertility caused by intrauterine fetal bone retention: a case report

**DOI:** 10.1186/1752-1947-8-177

**Published:** 2014-06-04

**Authors:** Songshu Xiao, Qi Tian, Min Xue

**Affiliations:** 1 Department of Gynecology and Obstetrics, Xiangya Hospital of Central South University, Changsha 410013, China

**Keywords:** Fetal bone retention, Hysteroscopy, Infertility

## Abstract

**Introduction:**

Intrauterine fetal bone retention is a rare complication and the bony fragments probably work like an intrauterine contraceptive device resulting in secondary infertility. Among the scarcely reported cases in the literature, there was no report described the retention of a large number of fetal bones with nearly intact morphology.

**Case presentation:**

The present report described an unusual case of fetal bone retention in a 30-year-old infertile Chinese woman who had a surgical termination of a 15-week pregnancy 9 years ago. The routine B-ultrasound diagnosed intrauterine foreign bodies. A hysteroscopy was performed which showed a large number of intrauterine bony fragments, with clear fetal skeletal outline and intact morphology. The detected residual fetal bones were removed under hysteroscopy, assisted by B-ultrasound scanning. The patient was pregnant 5 months later. The present case confirms the importance of routine examination of the intactness of the fetus after abortion, particularly when it happens in pregnancies of more than 12 weeks. Once diagnosed, the detected residual fetal bones should be removed by surgery, mainly under hysteroscopy.

**Conclusions:**

The retention of fetal bone may cause infertility, and removal of the residual bone may restore fertility. The improvement in hysteroscopy made it feasible to diagnose and remove the bones. The present case highlights the importance of examining the intactness of the removed fetus.

## Introduction

Intrauterine fetal bone retention is a rare complication and often occurs after abortion in the second and third trimesters [1]. The bony fragments probably work like an intrauterine contraceptive device (IUCD) to stimulate the secretion of endometrial prostaglandins, resulting in secondary infertility [2,3]. Hysteroscopic removal of the bony fragments may restore the fertility in most patients [4]. Among the scarcely reported cases in the literature, bony fragments were often observed in small pieces [1-5]. The present report described a rare case of infertility caused by the retention of a large number of fetal bones with nearly intact morphology.

## Case presentation

**Figure 1:**
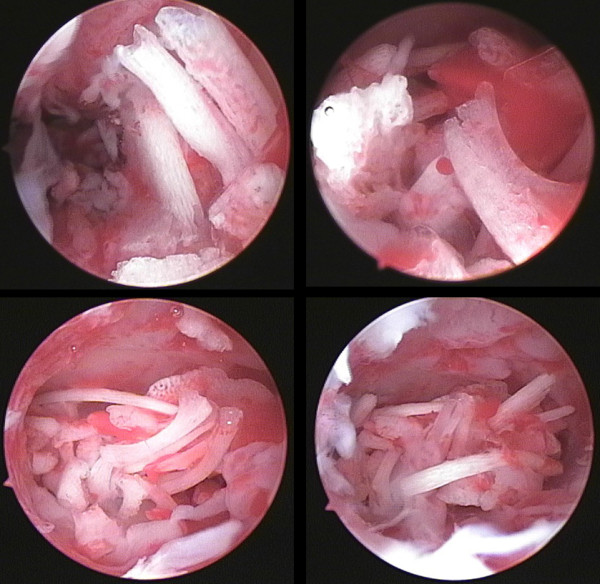
**A large number of strip-, bar-, rod-, and sheet-shaped pale fetal bones in the lower uterine segment can be observed under hysteroscopy, with clear outline and intact morphology.** The size of the thickest piece was approximately 20×10×3mm.

A 30-year-old G3P0 Chinese woman with a normal sexual life was infertile for 9 years after induction of labor in her 15th week of pregnancy in June 2002. The abortion procedure was unclear, but the patient felt no unusual discomfort at that time. Her menstrual cycle was 28 to 30 days, but her menstrual period was extended to 10 to 15 days, with medium volume and without dysmenorrhea. She did not perform any contraception, and except for infertility she was in a good health. Her husband’s semen was normal.A routine gynecological examination showed her reproductive system to be normal. However, a routine B-ultrasound indicated that her endometrium had an acoustic shadow of approximately 2.2×1.0×0.7cm in size at the uterine fundus level. A hysteroscopy was then performed and showed a uterine cavity depth of 6.5cm and a thin endometrium with rough surface and congestion. Her uterine cavity was filled with pale bone-like tissues in varying shapes and sizes. She was diagnosed with intrauterine fetal bone retention (Figure 1), and the residual fetal bones were then removed with forceps under hysteroscopy and B-ultrasound monitoring. The cleaning was performed until her uterine cavity became smooth. Her postoperative uterine cavity depth increased to 8.5cm. Pathological examination showed (in uterus) proliferative phase endometrium, mild chronic inflammation, and partial bone tissues.

She had a natural pregnancy 5 months after the surgery and delivered successfully and naturally in February 2013 (39 weeks and 3 days of pregnancy).

## Discussion

Intrauterine fetal bone retention is a rare complication; it mostly occurs after the induction of labor during second and third trimester pregnancy due to incomplete forceps delivery and curettage-resulted intrauterine retention of partial fetus [1,6], or it may be secondary, although rarely, to chronic inflammation-induced metaplasia [7]. Residual fetal bone often causes dysfunctional uterine bleeding, menorrhagia, dysmenorrhea, pelvic pain, abnormal vaginal discharge, infertility, and so on. [8]. In addition to the above clinical manifestations, it should be also noted that patients with fetal bone retention always have a history of induction of labor [5].

The rareness of the present case is that nearly complete fetal bone residues were observed under hysteroscopy; the large number and intact morphology of the residual bones are extremely rare among similar cases and, to the best of our knowledge, have never been reported previously [1-5]. The large number of residual fetal bones exerts an IUCD-like effect, causing secondary infertility. In this case, the residual fetal bones originated from an induction of labor in the 15th week of pregnancy 9 years ago. Although the bony fragments may cause multiple gynecological complaints, in this case the retention did not cause any symptoms except for an extended period which, to some extent, eventually delayed the treatment.

Once diagnosed, the detected residual fetal bones should be removed surgically under a B-ultrasound-monitored hysteroscopy [4]. The removal of residual fetal bones under hysteroscopy and B-ultrasound monitoring not only can ensure safety and reduce the occurrence of complications, but also the timely detection and removal of residual bones embedded in the uterine wall to guarantee the effectiveness of surgical treatment [9]. The present case indicates that in a termination of a pregnancy longer than 12 weeks, because of the formation of the fetus, the cervix should be fully softened and dilated prior to the operation and, after the surgery, the intactness of the removed fetus should be carefully examined. A review examination with B-ultrasound should also be performed for timely detection of any potential abnormalities.

## Conclusions

The retention of fetal bone may cause infertility, and removal of the residual bone may restore fertility. The improvement in hysteroscopy and the application of imaging technology made it feasible to diagnose and remove the bones and obtain clear intrauterine pictures. The operation should be conducted under ultrasound monitoring to ensure the removal of the retained fetal bones and to reduce the possibility of complications such as uterine perforation. The present case also highlights the importance of examining the intactness of the removed fetus, particularly when the abortion happened with a pregnancy longer than 12 weeks.

## Consent

Written informed consent was obtained from the patient for publication of this case report and any accompanying images. A copy of the written consent is available for review by the Editor-in-Chief of this journal.

## Competing interests

The authors declare that they have no competing interests.

## Authors’ contributions

SX participated in the design of diagnosis and treatment plan, surgical operation, and drafted the manuscript. QT consulted documents, organized patient data, and followed up the menstruation and pregnancy outcome. MX guided the manuscript draft and surgical operation. All authors read and approved the final manuscript.
